# The Effect of Oxandrolone Treatment on Human Osteoblastic Cells

**Published:** 2007-03-07

**Authors:** Lian Xiang Bi, Kristine M. Wiren, Xiao-Wei Zhang, Gisele V. Oliveira, Gordon L. Klein, Elgene G. Mainous, David N. Herndon

**Affiliations:** a Department of Oral and Maxillofacial Surgery, University of Texas Medical Branch, Galveston, TX; b Department of Pediatrics, University of Texas Medical Branch, Galveston, TX; c Department of Surgery, University of Texas Medical Branch, Galveston, TX; d Department of Orthopedic Surgery, University of Texas Medical Branch, Galveston, TX; e Department of Dermatology, University of Texas Medical Branch, Galveston, TX; f Shriners Burns Hospital, Galveston, TX; g Research Service, VA Medical Center, Portland, OR; h Departments of Medicine and Behavioral Neuroscience, Oregon Health and Science University, Portland, OR

## Abstract

**Objective:** Oxandrolone, administered to severely burned children over the first year postburn, produces increased lean body mass by 6 months; however, an increase in total body bone mineral requires 12 months. Consequently, this bone mineral response may be due to increased muscle mass. Alternatively, oxandrolone may act directly on bone. The current study seeks to determine whether oxandrolone can transactivate the androgen receptor in osteoblasts. **Methods:** Collagen, alkaline phosphatase, osteocalcin, osteoprotegerin, and androgen receptor abundance were determined by qRT-PCR, confocal laser scanning microscopy, or immunoquantitative assay. To determine the effect of oxandrolone on gene expression in differentiated cells, osteocytic cultures were grown to confluence in differentiation medium and then treated 24 hours or 5 days with 15 μg/mL oxandrolone. **Results:** Increased nuclear fluorescence of the androgen receptor and increased cellular type I collagen were observed with oxandrolone at 15 and 30 μg/mL but not at lower doses. Alkaline phosphatase (7%–20%) and osteocalcin (13%–18%) increases were modest but significant. Short-term treatment produced no significant effects, but at 5 days androgen receptor levels were increased while collagen levels were significantly decreased, with little effect on alkaline phosphatase, osteocalcin, or osteoprotegerin. **Conclusions:** These data suggest oxandrolone can stimulate production of osteoblast differentiation markers in proliferating osteoblastic cells, most likely through the androgen receptor; however, with longer treatment in mature cells, oxandrolone decreases collagen expression. Thus it is possible that oxandrolone given to burned children acts directly on immature osteoblasts to stimulate collagen production, but also may have positive effects to increase bone mineral through other mechanisms.

Long-term use of the orally administered anabolic agent oxandrolone has been shown to increase both lean body mass and bone mineral content in severely burned children when given over the first year postburn.^1^ Oxandrolone and recombinant human growth hormone^2^ were shown to be effective in recovering bone that would ordinarily be lost following the burn injury, resulting in markedly reduced bone formation^3^ hypocellularity at the mineralization front of bone^3^,^4^ and decreased marrow stromal cell differentiation into osteoblasts.^4^

Increased endogenous glucocorticoid production is likely responsible for the acute bone loss observed in severely burned patients.^3^,^4^ Notably, with both oxandrolone and recombinant human growth hormone, an increase in lean body mass precedes an increase in bone mineral content by 3 to 6 months.^1^,^2^ Thus it is not clear whether these anabolic agents increase bone mineral content secondary to increased muscle mass and hence increased skeletal loading, or whether they act directly on osteoblastic cells. Oxandrolone is an anabolic steroid with the ability to transactivate the androgen receptor (AR), which may be one mechanism underlying the anabolic response to therapy. The aim of our study is to determine whether oxandrolone can increase osteoblastic production of type I collagen and whether this action is mediated by AR signaling.

## MATERIALS AND METHODS

### Human osteoblast cell cultures

Freshly discarded human cancellous bones were obtained from healthy young patients undergoing osteotomy. The bone fragments were washed with serum-free α–minimum essential medium (α-MEM, Flow Laboratories, McLean, VA). Then, the fragments were digested in the medium with 1 mg/mL collagenase for 2 hours at 37°C. The enzymatic reaction was stopped by adding an equal volume of α-MEM with 10% fetal bovine serum (FBS). The supernatant containing the released cells was recovered. Washing and recovering were repeated 3 times. The cells were transferred to a centrifuge tube and centrifuge 10 minutes at 100 × *g* to harvest the cells. The cell pellet was resuspended in 5 mL of fresh medium. The single cell suspension was cultured in α-MEM containing antibiotics [penicillin (100~U/mL), streptomycin sulphate (100 μg/mL)] and 10% FBS in a humidified incubator at 37°C under an atmosphere of 5% CO_2_ and 95% air. After confluence, adherent cells were collected by trypsinizing with 0.025% trypsin-EDTA and resuspended in α-MEM. Only passages 4 to 8 were used in this study. Medium was changed every other day.^4^

### Osteocytic cell culture

Osteoblasts in vitro progress through several developmental stages that correlate with osteoblast development in vivo: from committed preosteoblasts to mature, differentiated osteoblasts, and finally to osteocyte-like cells embedded in mineralized extracellular matrix.^5^–^7^ To characterize the effects of oxandrolone in osteocytic cells, human osteoblast cells were cultured in α-MEM with 5% fetal bovine serum containing antibiotics for 10 days until confluence, then switched to BGJ_b_ medium containing 50 μg/mL ascorbic acid and 3 mM β-glycerol phosphate.^7^ Cultures were maintained in differentiation medium for 7 days for mature osteoblast/osteocyte development,^7^ then treated with oxandrolone for time course and dose-response analysis. These highly confluent osteocytic cultures were treated in 5% charcoal-stripped serum with either 15 μg/mL of oxandrolone for 24 hours or 5 days, or with increasing concentrations of oxandrolone (0, 1, 5, 10, and 15 μg/mL) for 5 days. Total RNA was isolated for gene expression analysis as described below.

### Immunohistochemistry for collagen type I and AR

Immunohistochemical staining was carried out for both the AR^8^,^9^ and type I collagen. Human osteoblastic cells were trypsinized, seeded on slide chambers (Lab-Tek, Nalge Nunc International, Rochester, NY) at a concentration of 1 × 10^5^ cells/mL and cultured for 3 days until they achieved 60% to 70% confluence. Oxandrolone was used to stimulate the cells at the concentration of 1, 5, 10, 15, and 30 μg/mL for 24 hours. Stock solution containing 2.5~mg diluted in 2 mL of DMSO was prepared and subsequently diluted in culture media, while control nonstimulated cells received the same solution without oxandrolone. Thereafter, the medium was discarded, the cells were washed with phosphate buffered saline (PBS), and fixed in −20°C methanol/acetone (50:50) for 20 minutes and permeabilized with 0.5% Triton X-100 (Sigma, St Louis, MO). The polyclonal antibody used for collagen I was applied overnight at the dilution of 1:200 (Research Diagnostics Inc, Flanders, NJ). The polyclonal antibody for AR (Neomarkers, Fremont, CA) was used at the dilution of 1:30. The next day, FITC-conjugated goat anti-rabbit IgG was used as secondary antibody (Neomarkers) and cell nuclei were stained with 4,6-diamidino-2-phenylindole (DAPI) (Vector Mounting Medium, Burlingame, CA). Images were captured using the laser confocal scanning microscope (Zeiss LSM 510, Jena, Germany) with a 63 × objective. Optical sections were obtained from the cells by capturing single images of central cell focal planes. Three microscopic fields were captured for the control group of each cell line and the same was done for cells treated with oxandrolone. To proceed to an analysis of AR immunohistochemistry, the intensity of fluorescence was measured in the nuclei (AR) in 2 cells per 3 fields, using Image Tool software (University of Texas Health Science Center, San Antonio).

### Assay of alkaline phosphatase

Human osteoblastic cells were cultured in α-MEM containing antibiotics and 10% FBS. The cells were challenged with increasing concnetrations of oxandrolone (0, 1, 5 10, 15 μg/mL). After 24 hours of treatment, the levels of alkaline phosphatase activity were determined using a commercial kit (Pierce Biotechnology, Inc, Rockford, IL). The cells were washed with cold PBS and subjected to 3 freeze-thaw cycles. These samples were assayed for enzymatic activity with *p*-nitrophenyl phosphate as a substrate. Sample absorbance was measured at 400 nm with microplate reader. Results were expressed in ALP (OD)/protein (OD).

### Immunoquantitative assay for collagen type I and osteocalcin

To perform the immunoquantitative assay for type I collagen^4^ and osteocalcin, human osteoblastic cells were subcultured on 96-well plates until they achieved 70% confluence. Oxandrolone was used to stimulate the cells at the concentration of 0, 1, 10, 15, and 30~μg/mL for 24 hours. Cells were harvested and washed with cold PBS, fixed in methanol for 10 minutes at −20°C and washed with PBS. They were then preincubated with primary antibody for type I collagen and osteocalcin (Santa Cruz Biotechnology Inc, CA), respectively, overnight at 4°C, followed by secondary biotinylated IgG for 30 minutes at room temperature. After washing with PBS, cells were exposed to *p*-nitrophenyl phosphate containing levamisole to inhibit the generation of *p*-nitrophenol by endogenous alkaline phosphatase for 8 minutes. Sample absorbance was measured at 400 nm with an ELISA reader. The values (OD) were normalized with protein concentration (OD).

### Real-time quantitative reverse transcription–polymerase chain reaction (qRT-PCR) on cultured human osteocytic cells

Total RNA was isolated from osteocytic cultures and gene expression characterized by qRT-PCR analysis using human primers from Qiagen (Valencia, CA) specific for AR and the osteoblast marker proteins alkaline phosphatase, type I collagen, osteocalcin, and osteoprotegerin. The qRT-PCR analysis was performed with the iCycler IQ Real Time PCR detection system (Bio-Rad Laboratories, Inc, Hercules, CA) using a one-step QuantiTect SYBR Green RT-PCR kit (Qiagen) on DNase-treated total RNA. Relative expression of the RT-PCR product was determined using the comparative ΔΔ *C*_t_ method after normalizing expression with fluorescence to the specific RNA-binding dye RiboGreen (Molecular Probes, Eugene, OR) as previously described.^10^ Real-time qRT-PCR efficiency was determined for each primer set using a fivefold dilution series of total RNA and did not differ significantly from 100%. Individual reaction kinetics were also analyzed to ensure each real-time RT-PCR did not differ significantly from 100% efficiency. Following PCR, specificity of the PCR reaction was confirmed with inverse derivative melt curve analysis. Data is presented as mean ± SEM.

### Statistical analyses

Data are reported as mean ± SEM. The statistical significance of intergroup differences was tested using the Student *t* test when the variances were equal. *P* less than .05 was considered significant.

## RESULTS

### Oxandrolone treatment in proliferating human osteoblastic cells

**Figure 1:**
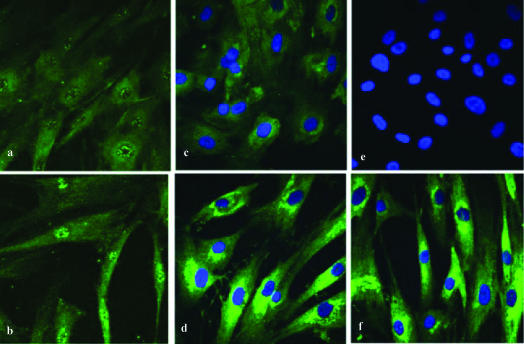
Confocal scanning laser microscopic analysis of human osteoblastic cells after oxandrolone treatment. (a) Confocal scanning laser microscopy depicting AR nuclear fluorescence of human osteoblastic cells not stimulated with oxandrolone, (b) confocal scanning laser microscopy depicting the increased AR nuclear fluorescence of human osteoblastic cells stimulated with oxandrolone 30 μg/mL, (c) confocal scanning laser microscopy depicting cytoplasmic fluorescence of type I collagen in human osteoblastic cells not stimulated with oxandrolone, (d) confocal scanning laser microscopy depicting increased cytoplasmic fluorescence of type I collagen in human osteoblastic cells stimulated with oxandrolone 30 μg/mL, (e) internal negative control for primary antibodies of AR and type I collagen, (f) confocal scanning laser microscopy depicting increased cytoplasmic fluorescence of type I collagen in human osteoblastic cells stimulated with oxandrolone 15 μg/mL. Compare to Figures 1c and 1d.

Proliferating cultures of normal human osteoblastic cells were treated for 24 hours with 30 μg/mL oxandrolone or vehicle to determine the acute response to oxandrolone treatment. Although the affinity for oxandrolone for AR is approximately 100-fold lower than testosterone, oxandrolone treatment still results in AR transactivation.^11^ To characterize the ability of oxandrolone to stimulate translocation of the AR to the nucleus, confocal laser scanning microscopy was employed. When control cultures were stained for AR, diffuse cytoplasm staining but only weak nuclear staining were observed on osteoblasts nonstimulated with oxandrolone (Fig 1a). After stimulation with oxandrolone, increased fluorescence of AR in the nuclei was seen (Fig 1b), suggesting transactivation by oxandrolone at these concentrations.

We next assessed the effect of oxandrolone treatment on type I collagen, the major constituent of the bone matrix. Immunohistochemistry for collagen type I on cultured osteoblast cells demonstrated an increase in collagen that was dose dependent. When compared to control group (Fig 1c), a significant increased intensity of fluorescence was seen for cells stimulated with oxandrolone. This was observed when the concentration of oxandrolone was 15 μg/mL (Fig 1f) and 30 μg/mL (Fig 1d).

**Figure 2:**
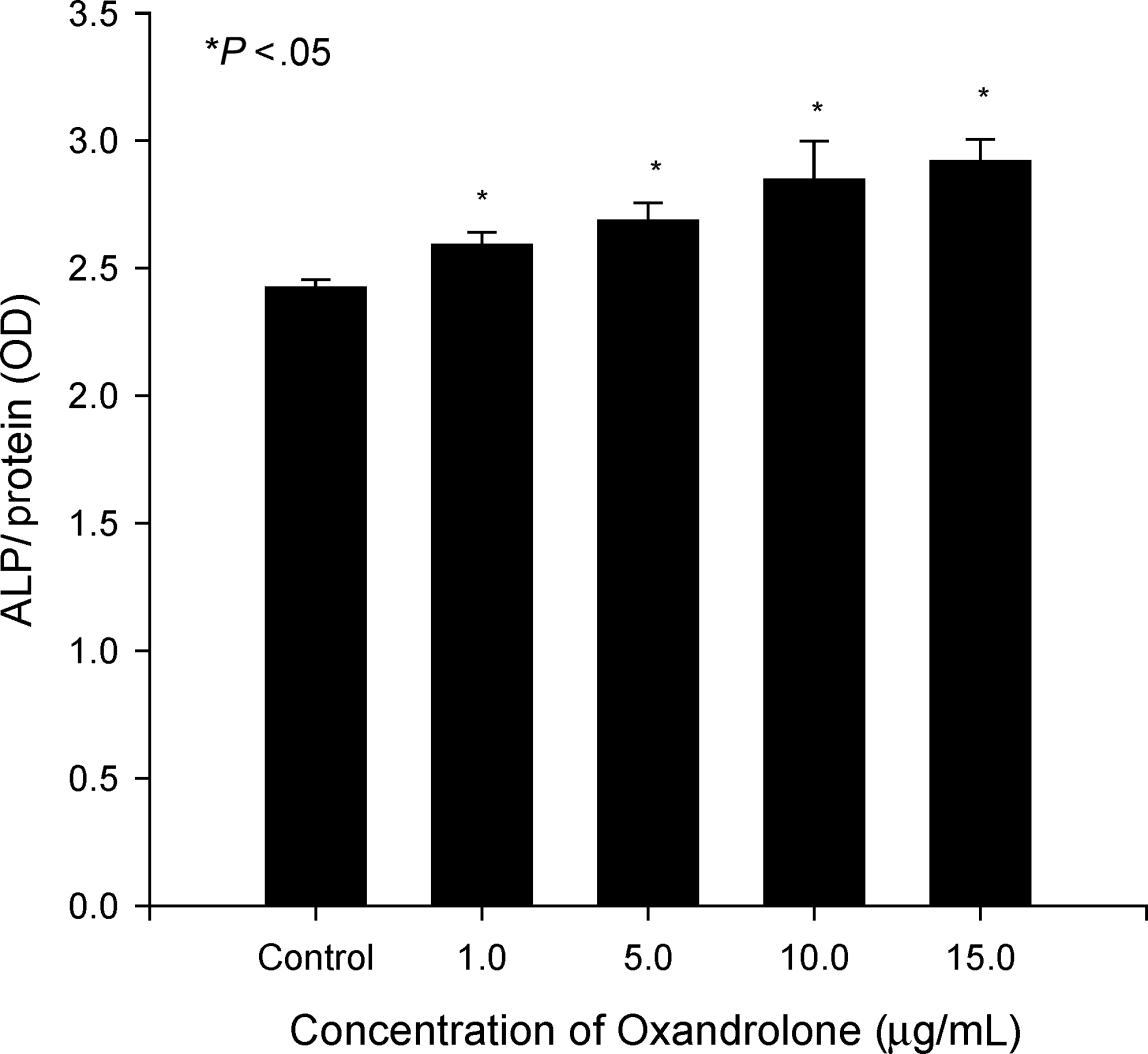
Stimulation of alkaline phosphatase activity by oxandrolone in proliferating human osteoblastic cultures. Proliferating cultures were treated acutely for 24 hours with 0, 1, 5, 10, and 15 μg/mL oxandrolone. Data are expressed as mean ± SEM of 6 determinations.

**Figure 3:**
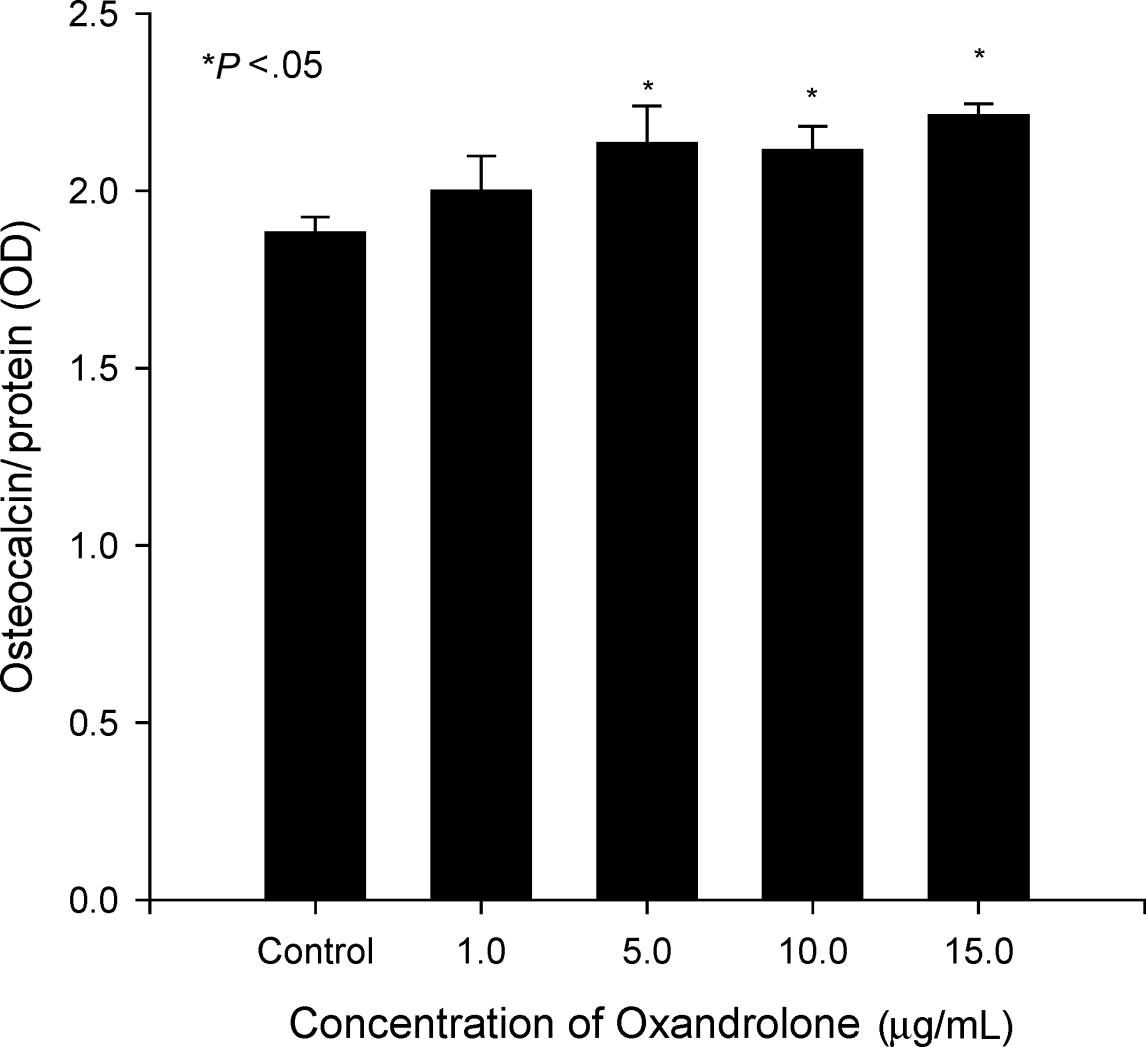
Increased osteocalcin levels after oxandrolone treatment. Human osteoblastic cultures were exposed to oxandrolone (0, 1, 5, 10, and 15 μg/mL) for 24 hours. Data are expressed as mean ± SEM of 6 determinations.

To determine the effect of oxandrolone on additional markers of osteoblast activity, osteoblastic cultures were treated with or without oxandrolone. Alkaline phosphatase activity was determined in cells treated with oxandrolone for 24 hours (Fig 2), and there was an increase (7%–20%) in activity in a dose-dependent manner. The differences were statistically significant (*P* < .05) but the changes were minor compared to the control group. We also performed immunoquantitative assays for type I collagen and osteocalcin on osteoblastic cells. In cultures treated with oxandrolone (10 or 15 μg/mL), collagen was increased 21% to 35% (Fig 3; *P* < .05). There were no differences in the groups treated with low concentrations of oxandrolone (1 or 5 μg/mL) compared to control group. Thus, after multiple repetitions of the experiment, the data confirmed the results observed using immunofluorescence.

**Figure 4:**
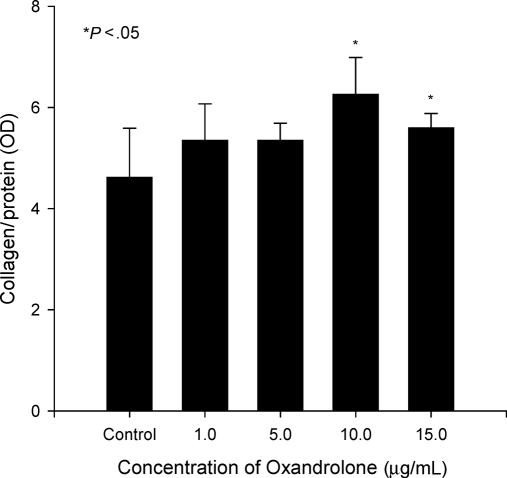
Enhanced type I collagen secretion after oxandrolone exposure. Proliferating cultures were treated with 0, 1, 5, 10, and 15 μg/mL oxandrolone for 24 hours. Data are expressed as mean ± SEM of 6 determinations.

We next assessed the levels of osteocalcin, an important biochemical indicator of bone turnover.^12^ Cells treated with oxandrolone of 10, 15, and 30 μg/mL produced a greater level of osteocalcin (11%–18%) compared to the control group. The differences were statistically significant (Fig 4; *P* < 0.05) but again the increase was small. Thus, the production of osteocalcin mirrored the pattern of alkaline phosphatase activity.

**Figure 5:**
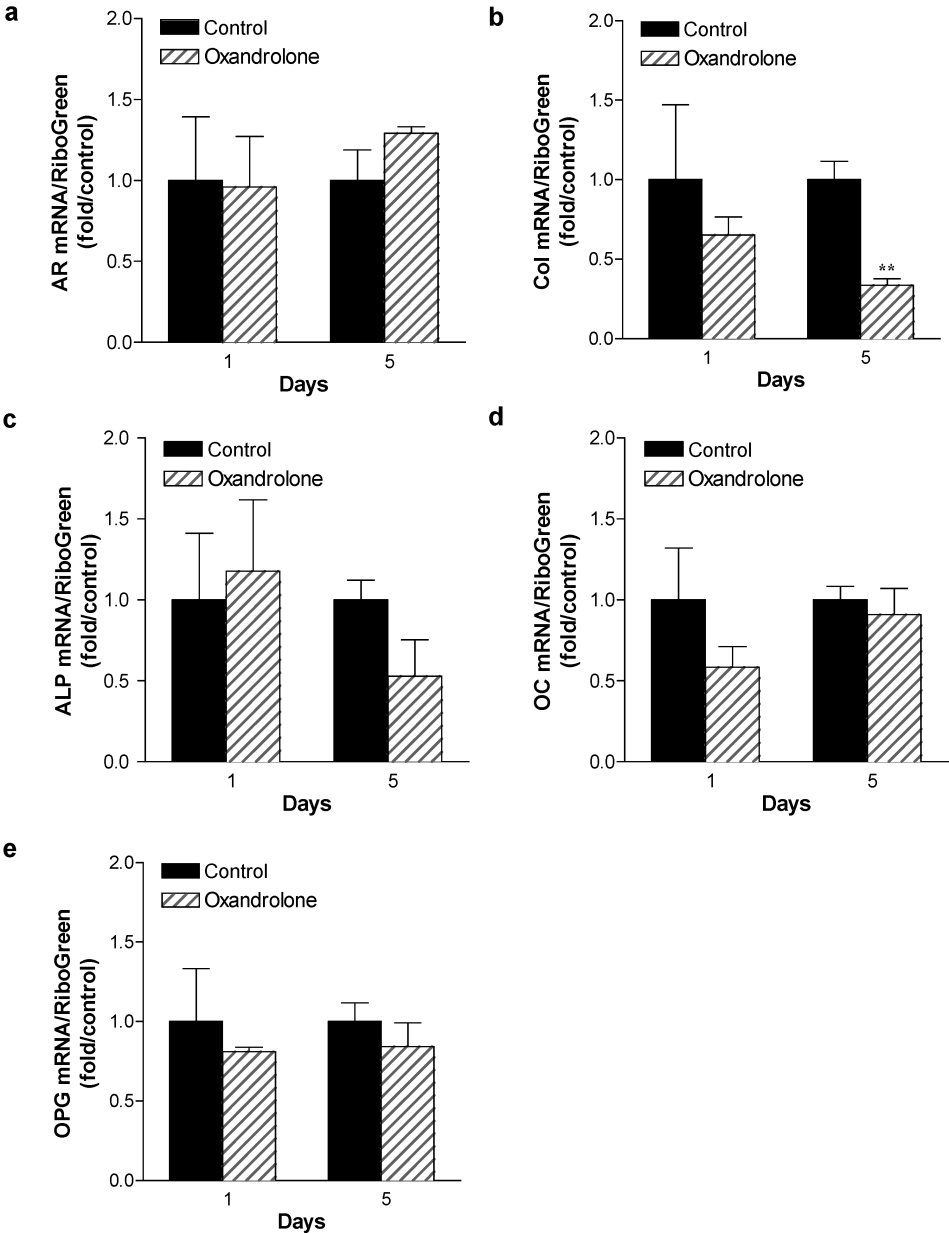
Consequence of oxandrolone treatment on gene expression in normal human osteocytes: time course analysis. Normal human osteoblastic cells were cultured for 10 days to confluence, then switched to differentiation medium containing ascorbic acid and β-glycerol phosphate. After 7 days, osteocytic cells were treated with 15 μg/mL oxandrolone. Total RNA was isolated after 24 hours or 5 days treatment, and gene expression characterized by qRT-PCR analysis using human primers specific for AR, type I collagen (col), alkaline phosphatase (ALP), and osteocalcin (OC) and osteoprotegerin (OPG). *n* = 3 to 4. **P < .01.

**Figure 6:**
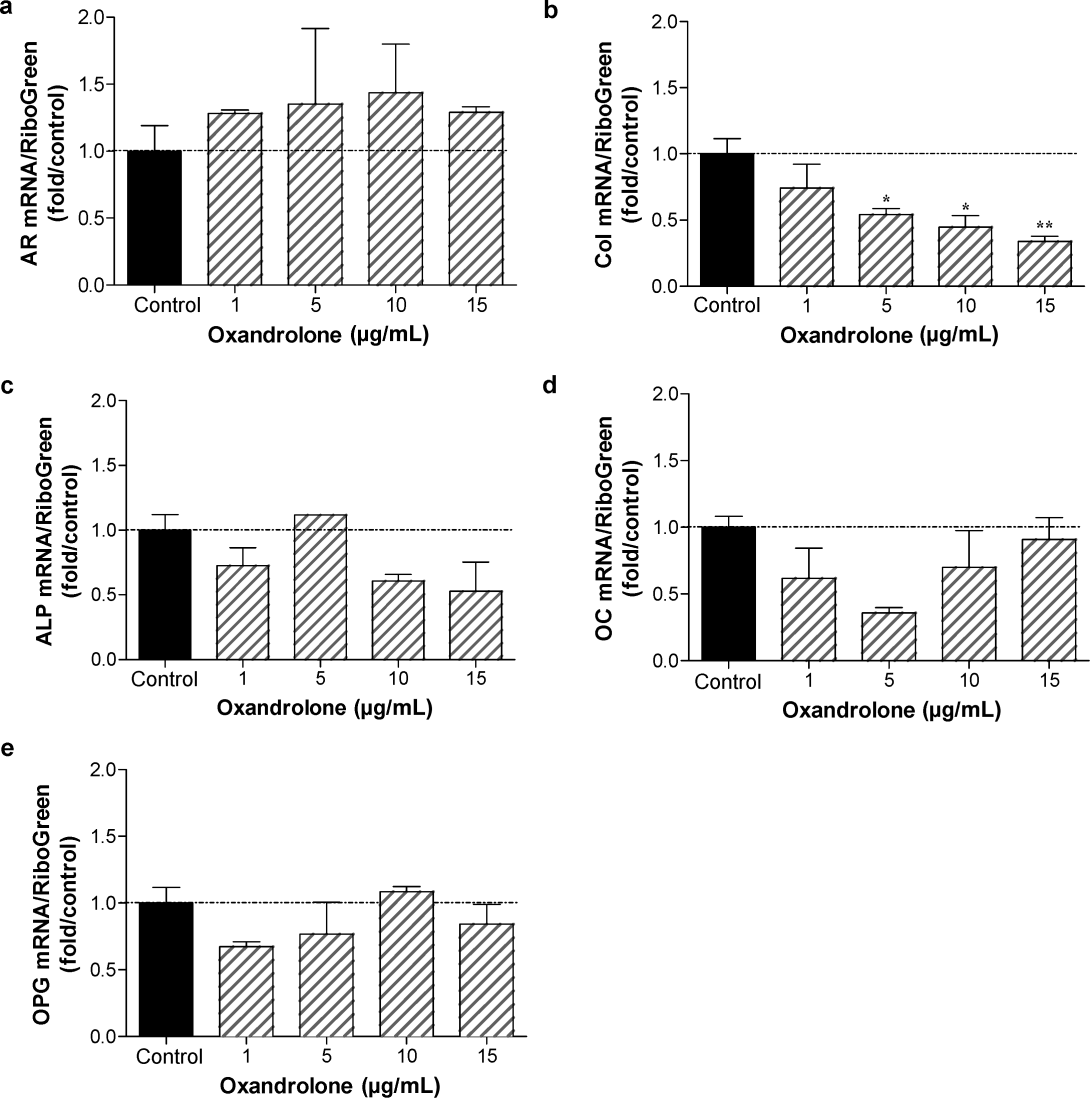
Consequence of oxandrolone treatment on gene expression in normal human osteocytes: dose-response analysis. Normal human osteoblastic cells were cultured as described in Figure 5. Osteocytic cultures were treated for 5 days with 0, 1, 5, 10, and 15 μg/mL oxandrolone. Total RNA was isolated and gene expression characterized by qRT-PCR analysis using human primers specific for AR, type I collagen (col), alkaline phosphatase (ALP), osteocalcin (OC) and osteoprotegerin (OPG). *n* = 2 to 4. *P < .05; **P < .01.

AR concentrations increase as osteoblasts differentiate, reaching their highest levels in osteocytic cultures.^13^ Furthermore, the most abundant cell in bone is the osteocytic cell. We therefore determined the effect of oxandrolone treatment on gene expression in mature osteocytic cultures. Normal human osteoblasts were cultured for 10 days, then switched to differentiation medium containing ascorbic acid and β-glycerol phosphate. After 7 days, osteocytic cells were treated with 15 μg/mL oxandrolone. Total RNA was isolated after 24 hours or 5 days treatment, and gene expression characterized by qRT-PCR analysis using human primers specific for AR, type I collagen, alkaline phosphatase, osteocalcin, and osteoprotegerin (Fig 5). Although there was little effect of oxandrolone after 24 hours of treatment in osteocytic cells on type I collagen, alkaline phosphatase, osteocalcin, or osteoprotegerin mRNA abundance, a nonsignificant increase in AR mRNA was noted. This is consistent with other reports documenting an increase in AR after androgen treatment in osteoblasts.^14^ After stimulation with oxandrolone for 5 days, a significant decrease in expression of type I collagen was seen (*P* < 0.01). Thus, longer treatments with oxandrolone in osteocytic cultures reduced collagen expression. These data are consistent with dose-response studies performed after 5 days of treatment with increasing concentrations of oxandrolone (Fig 6), where AR mRNA is modestly increased but collagen levels are significantly decreased in a dose-dependent fashion with oxandrolone treatment.

## DISCUSSION

In this study we have shown that stimulation of cultured human osteoblasts by oxandrolone results in immunofluorescent detection of AR in the nucleus and increased osteomarkers in these osteoblasts. These data suggest that oxandrolone directly targets human osteoblasts by means of the AR, resulting in increased expression of osteoblast differentiation markers after short-term treatment. Therefore, oxandrolone may act directly on the osteoblast in addition to effects that result in increasing skeletal loading.

Immunohistochemistry and confocal laser scanning microscopy (CLSM) were used to evaluate the expression of AR and type I collagen in osteoblasts, with several advantages over regular fluorescence microscopy.^15^,^16^ While in conventional wide-field fluorescence microscopy the emitted light coming from regions above and below the focal plane is collected by the objective lens and contributes to an out-of-focus blur in the final image, in the CLSM a diaphragm rejects the out-of-focus information.^15^,^16^ Therefore, tissue thickness does not interfere with the resulting fluorescence. The increased expression of type I collagen by osteoblasts was also determined by immunoassay confirming the results observed with immunohistochemistry.

The AR is a steroid receptor that generally mediates biologic responses to androgens. The increased expression of AR in the present study is consistent with that documented in the literature.^14^ In bone tissue the AR is expressed in a variety of cell types, but its specific role in the maintenance of skeletal homeostasis remains controversial.^17^ In an experimental study, androgen deficiency was shown to result in a substantial loss of cancellous bone in the axial and appendicular skeleton of aged male rats and that this osteopenia is associated with a sustained increase in bone turnover.^18^ Consistent with this, the bone phenotype that develops in a global AR null (ARKO) male mouse model is a high-turnover osteopenia, with reduced trabecular bone volume, and a significant stimulatory effect on osteoclast function.^19^,^20^

Alkaline phosphatase is well recognized as a marker that reflects osteoblastic activity.^21^ Kasperk et al reported that androgens increase the alkaline phosphatase activity in osteoblast culture.^22^–^24^ However, there are also reports of androgens either inhibiting^25^ or having no effect on alkaline phosphatase activity,^26^,^27^ which may reflect both the complexity of osteoblastic differentiation and the various model systems employed. In a clinical study, Murphy et al^1^ have shown that oxandrolone administration increases levels of serum alkaline phosphatase in treated patients by 6 months versus controls. In the present study, the cells treated with oxandrolone produced a greater level of alkaline phosphatase. The elevated activity of this cellular enzyme suggests an early increase in the osteoblast differentiation process.

Osteocalcin appears to be a bone-specific gla-containing protein, accounting for 10% to 20% of the noncollagenous protein in bone. While the in vivo function of osteocalcin remains unclear, its affinity for bone mineral constituents implies a role in bone formation. Hence it has been shown that osteocalcin is a biochemical indicator of bone turnover.^12^ In our study, proliferating cells treated for 24 hours with oxandrolone produced small but statistically significant increases in osteocalcin production compared to control group.

To test the effect of oxandrolone on osteocyte-like cells differentiated from osteoblasts at the molecular level, the human osteoblastic cells were cultured in a differentiation medium and then exposed to oxandrolone. The results showed that short-term treatment produced little effect on type I collagen, alkaline phosphatase, osteocalcin, osteoprotegerin, or AR mRNA. Long-term treatment decreased type I collagen expression, consistent with decreased collagen expression observed by Wiren et al^16^ in AR-transgenic mice with targeted AR overexpression in the osteoblast lineage.^16^ The results described here are consistent with observations reported in vivo with male AR-transgenic mice, where calvarial thickening is observed and cortical formation is altered with surface periosteal expansion but inhibition of inner endosteal deposition, consistent with the known effects of androgen to stimulate periosteal apposition. The dramatic inhibition at the endosteal envelope may be responsible for a modest decrease in cortical bone area and reductions in biomechanical properties observed.^16^ Thus, taking all data together, our results suggest that osteoblast cells are targeted by androgens to transactivate AR in bone.

Murphy et al^1^ have recently shown that oxandrolone administration increases lean body mass 3 to 6 months before an increase in bone mineral content is observed. On the other hand, we have found an increase in collagen production when high doses of oxandrolone were used to stimulate osteoblasts. The applicability of our findings in this study to the in vivo effects of oxandrolone in burned children^1^ is not clear. While an in vitro environment may not adequately reproduce the in vivo situation, many potential variables are eliminated during in vitro studies, and it is possible to investigate a specific effect of a drug on cells. The time frame of in vivo and in vitro settings is plainly different, and the concentration of oxandrolone used to stimulate cells in the in vitro setting is probably higher than the one used in the clinical trial.^1^ Therefore, the results may not be strictly comparable. Although this study demonstrates that oxandrolone is capable of affecting the osteoblast AR and stimulating type I collagen synthesis, regulatory mechanisms that are present only in a complex in vivo situation may account for increased collagen turnover or degradation after synthesis.

Moreover, the delay in the increase of total body bone mineral content reported in the clinical study by Murphy et al^1^ may be explained in part by the acute inhibition of bone formation and osteoblast differentiation after a severe burn as previously reported.^3^,^4^ Thus there may have been a lack of osteoblasts, and therefore osteoblast AR, to mediate a response to oxandrolone.

In conclusion, the use of high-dose oxandrolone results in increased nuclear fluorescence of the osteoblast AR, increased osteoblast differentiation markers in cultured osteoblasts and decreased bone marker in cultured osteocyte-like cells in vitro. Oxandrolone may have the ability to directly stimulate bone collagen synthesis, over and above its effect on skeletal loading effected through an increase in lean body mass following long-term treatment. However, longer exposure may no longer be directly anabolic in mature bone cells. This observation may help to explain the variability and confusion regarding androgen actions on the skeleton.
